# Radiopaque drug-eluting embolisation beads as fiducial markers for stereotactic liver radiotherapy

**DOI:** 10.1259/bjr.20210594

**Published:** 2021-11-16

**Authors:** Laura Beaton, Mairead Daly, Henry FJ Tregidgo, Helen Grimes, Syed Moinuddin, Chris Stacey, Sami Znati, Julian Hague, Zainab A Bascal, Paul E Wilde, Sarah Cooper, Steven Bandula, Andrew L Lewis, Matthew J Clarkson, Ricky A Sharma

**Affiliations:** 1 University College London Cancer Institute, University College London, London, United Kingdom; 2 University College London Hospitals NHS Foundation Trust, London, United Kingdom; 3 Department of Medical Physics and Biomedical Engineering, University College London, London, United Kingdom; 4 Biocompatibles UK Ltd, Lakeview, Riverside Way, Watchmoor Park, Camberley, Surrey, United Kingdom; 5 National Institute for Health Research University College London Hospitals Biomedical Centre, University College London Cancer Institute, London, United Kingdom

## Abstract

**Objective:**

To determine the feasibility of using radiopaque (RO) beads as direct tumour surrogates for image-guided radiotherapy (IGRT) in patients with liver tumours after transarterial chemoembolisation (TACE).

**Methods:**

A novel vandetanib-eluting RO bead was delivered via TACE as part of a first-in-human clinical trial in patients with either hepatocellular carcinoma or liver metastases from colorectal cancer. Following TACE, patients underwent simulated radiotherapy imaging with four-dimensional computed tomography (4D-CT) and cone-beam CT (CBCT) imaging. RO beads were contoured using automated thresholding, and feasibility of matching between the simulated radiotherapy planning dataset (AVE-IP image from 4D data) and CBCT scans assessed. Additional kV, MV, helical CT and CBCT images of RO beads were obtained using an in-house phantom. Stability of RO bead position was assessed by comparing 4D-CT imaging to CT scans taken 6–20 days following TACE.

**Results:**

Eight patients were treated and 4D-CT and CBCT images acquired. RO beads were visible on 4D-CT and CBCT images in all cases and matching successfully performed. Differences in centre of mass of RO beads between CBCT and simulated radiotherapy planning scans (AVE-IP dataset) were 2.0 mm mediolaterally, 1.7 mm anteroposteriorally and 3.5 mm craniocaudally. RO beads in the phantom were visible on all imaging modalities assessed. RO bead position remained stable up to 29 days post TACE.

**Conclusion:**

RO beads are visible on IGRT imaging modalities, showing minimal artefact. They can be used for on-set matching with CBCT and remain stable over time.

**Advances in knowledge:**

The role of RO beads as fiducial markers for stereotactic liver radiotherapy is feasible and warrants further exploration as a combination therapy approach.

## Introduction

Stereotactic body radiation therapy (SBRT), or stereotactic ablative body radiosurgery (SABR), is an advanced radiotherapy technique which uses precise targeting to deliver high dose, highly focussed external beam radiotherapy in a small number of fractions (usually 1–5). SBRT is a feasible and safe therapeutic option for patients with hepatocellular carcinoma (HCC) ineligible for other local treatments, with studies showing local control rates of 70–100%, comparable to that of radiofrequency ablation RFA.^1–9^ SBRT can also be used safely in the adjuvant setting and as a salvage treatment following transarterial chemoembolisation (TACE).^10–12^ It has been shown that patients who received SBRT following incomplete TACE had a 2-year survival rate that was significantly higher compared to those that received repeat TACE alone (36.8% *vs* 14.3%, *p* = 0.001).^13^ SBRT for liver oligometastases is also an effective treatment option for patients with advanced colorectal cancer, with encouraging local control and survival rates.^14,15^ In the UK, SBRT has now been commissioned for use in both HCC and liver oligometastases.

The doses delivered in SBRT are ‘ablative’ and accordingly, target accuracy is crucial for liver SBRT due to the radiosensitive nature of the liver, proximity of tumours to the small bowel and significant liver motion with respiration. However, liver tumours are often difficult to visualise on radiotherapy planning computed tomography (CT) scans without the use of contrast. Image-guided radiotherapy (IGRT) is therefore typically accomplished through visualisation of a surrogate to the tumour. Surrogates can be either the whole liver, diaphragm or through the insertion of specific fiducial markers (such as commercially available gold seeds). Fiducial markers implanted close to a liver tumour have been shown to be a better surrogate of tumour position than adjacent anatomical landmarks.^16^

TACE involves the introduction of a chemotherapeutic drug into arteries feeding a liver tumour, followed by an embolic agent that causes vessel stasis. Gelatin sponge and polyvinyl alcohol particles have been popular particulate embolic agents for TACE, whilst the embolic agent lipiodol has the advantage of being radiopaque on CT imaging. As such, lipiodol has already been shown to be a feasible surrogate for liver position during SBRT.^17,18^ More recently, cytotoxic agents have been combined with microspheres in order to create drug-eluting bead TACE (DEB-TACE). The DEBs, DC Beads^™^ (BTG, United Kingdom), are non-biodegradable polyvinyl alcohol microspheres, that can be loaded with calibrated cytotoxic drugs. The recent development of novel radiopaque (RO) drug-eluting beads, which can be visualised on CT or fluoroscopic imaging,^19^ has the advantage of providing intra- and post-procedure visualisation of the tumour vasculature. Furthermore, the durable radiopacity means that RO drug-eluting beads are visible on x-ray-based imaging after treatment.^20,21^ The retention of RO beads within the tumour vasculature means that RO drug-eluting beads could potentially act as a surrogate for direct image-guided tumour targeting.^18^

The aim of this study was to determine the feasibility of using RO drug-eluting beads as direct tumour surrogates (fiducial markers) for IGRT in patients with liver tumours post-TACE. Specifically, this study focussed on the simulated radiotherapy planning dataset (AVE-IP images from 4D CT data) and cone-beam CT (CBCT) images acquired following treatment with a novel vandetanib-eluting RO bead (VERB), in a first-in human clinical trial (the VEROnA trial).^22^ The VERB is a pre-loaded drug-eluting RO bead measuring 60–160 µm diameter. An in-house phantom model was also used to assess the visibility of the RO beads in additional imaging modalities required for IGRT, including kV, MV, helical CT and CBCT.

## Methods and materials

### Patient selection

**Figure 1.**
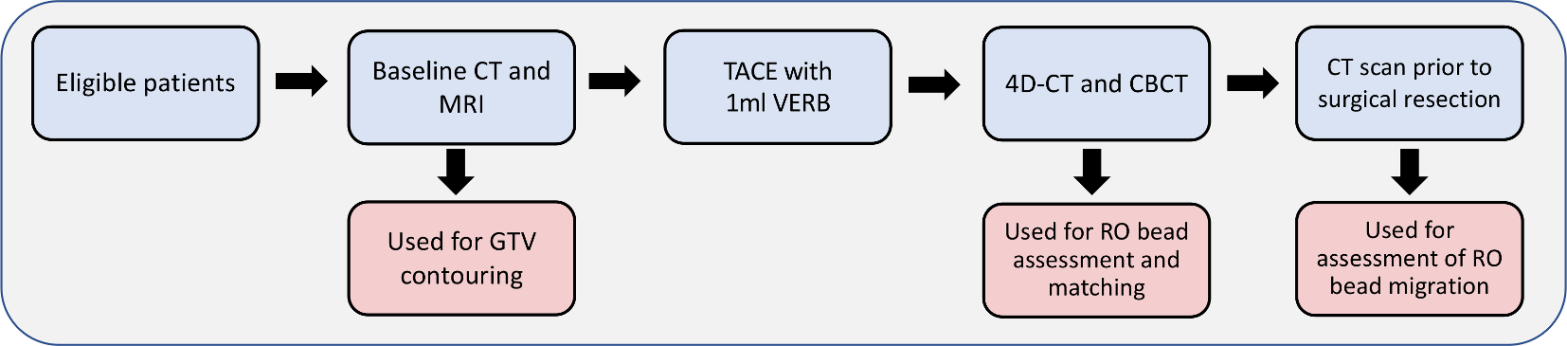
Basic overview of trial schema. All eligible patients were treated with 1 ml VERB (containing 100 mg vandetanib) via TACE, 7–21 days prior to surgical resection

The VEROnA study was a pilot, open-label, single-arm, phase 0, window-of-opportunity trial of VERB delivered transarterially, 7–21 days prior to surgery in patients with resectable liver malignancies (Figure 1). Eligible patients had a diagnosis of liver-limited colorectal metastases (mCRC), or HCC (Child-Pugh A), diagnosed histologically or radiologically, and were candidates for surgical resection. All patients were treated with up to 1 ml of VERB, containing 100 mg of vandetanib. The end point of the procedure was either full delivery of the reconstituted bead volume or near-stasis in the tumoural vessel. As part of this clinical trial, all patients underwent imaging with simulated radiotherapy planning scans (4D-CT) and CBCT 24 h following treatment with VERB. Furthermore, patients underwent diagnostic imaging with CT and magnetic resonance imaging (MRI) pre- and post-TACE with VERB.^22^

### 4D-CT and CBCT acquisition details

For acquisition of the simulated radiotherapy planning scans (4D-CT), all patients were positioned supine with arms up on a wing board and immobilised using a commercial immobilisation system (Combifix^™^). Patients were instructed to breathe normally and abdominal compression was not used. The Varian Respiratory Position Management (RPM) system was used to generate a surrogate respiration signal from a marker block placed on the patient’s chest. For each patient, 1.25 mm slice thickness 4D-CT images (120kV 100mA) were acquired on a GE Lightspeed CT scanner (Chicago, Illinois). The images were phase-sorted into ten bins of equal time using the surrogate respiration signal from the Varian RPM. The AVE-IP data set was then primarily used for analysis. Following this, each patient was set-up in the same position, using reference skin marks and a three-point laser system on a TrueBeam linear accelerator (Varian Medical Systems, Palo Alto, CA). A free-breathing CBCT scan was acquired using the on-board imaging system (125 kV, 60 mA, 20 ms, 1080 mAs). The reconstructed image volume was 17 cm along the cranio-caudal axis and 45 cm in the axial plane. A reconstructed slice thickness of 2.5 mm was used. It is important to highlight that patients in this study did not undergo radiotherapy treatment.

### RO bead evaluation

In order for RO beads to be used as fiducial markers for liver SBRT, they need to be visible at radiotherapy treatment planning (on the simulated planning data set, AVE-IP scan), visible on the CBCT (used for treatment verification), have the same shape and size (volume) on the simulated planning dataset and CBCT and have the same spatial separation in order to allow reliable matching, and finally show stability with negligible migration. Furthermore, in keeping with current recommendations, it was decided that the following criteria should apply: that the volume of the RO bead area needed to be >0.1 cm^3^ (based on the size of commercial fiducials), that the RO bead area needed to be within 4 cm of the GTV^16^ and that a minimum of three discrete RO beads areas would be identified.

**Figure 2.**
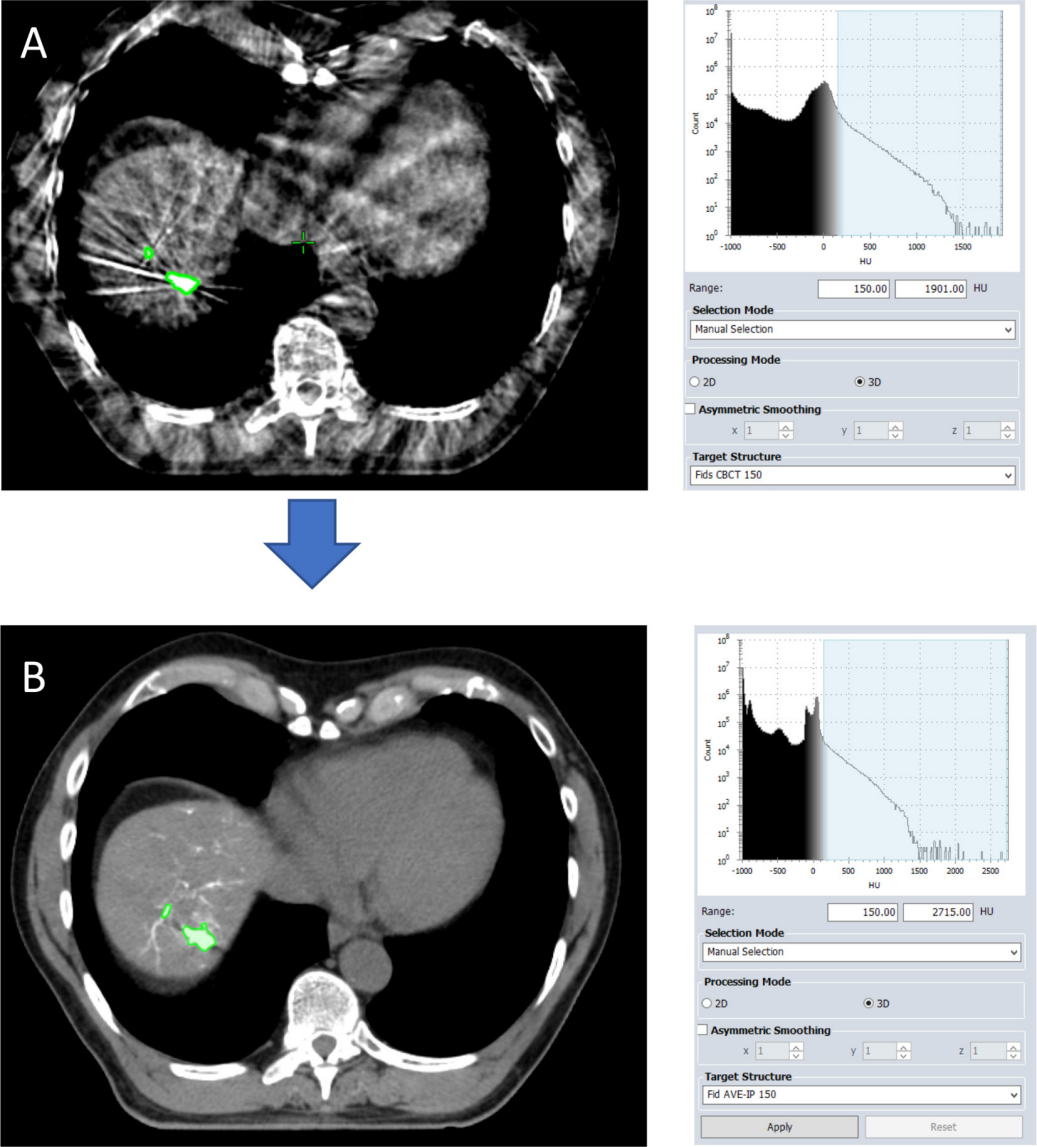
Automated threshold contouring on the CBCT and AVE-IP scans. Areas of beads with a denisty above 150 HU are automatically contoured on the cone-beam CT (CBCT) scan (image A) and average-intensity CT (AVE-IP) scans (image B)

RO bead areas were initially contoured on the CBCT scan by a certified radiation oncologist. As the RO individual beads typically measure 60–160 µm, they are deposited and align along liver blood-vessels and there are not clearly delineated regions of hyper-intensity, as seen with commercial fiducials (such as gold seeds) or Lipiodol. As such, in order to avoid inter-observer variation, the automated thresholding function in the Eclipse treatment planning system (Varian Medical Systems, Paolo Alto, CA) was utilised for contouring regions of hyperintensity where the beads had clumped together and were more visible as conglomerates. Automated thresholding automatically detects areas above a set Hounsfield unit (HU) threshold (Figure 2). Firstly, a lower HU threshold limit was selected. As these were novel RO beads that were being assessed for the first time on radiotherapy planning images, in order to define the lower limit, the CBCT for each patient was reviewed, and the three regions most visible as discrete areas selected and manually contoured. The mean HU of these volumes was chosen as the initial threshold and subsequent increments of 50 HU made. Automated thresholding was subsequently used to detect all areas with a HU value greater than the lower limit selected within the liver. Using the HU thresholds from the CBCT data, the same contouring approach was then applied to the average intensity projection (AVE-IP) scan of the 4D-CT, which was used as the primary radiotherapy planning data set. Gross tumour volume (GTV) was contoured for each treated tumour by a radiation oncologist, using fused baseline MRI and CT scans taken prior to TACE. All RO bead areas >4 cm away from the GTV were excluded.^16^ Any discrete volumes measuring <0.1 cm^3^ were removed (Supplementary Appendix A1). Finally, for visual comparison only, this process was repeated on the maximum intensity projection (MIP) scan from the 4D-CT data set.

Supplementary Material.

### Matching of RO beads from AVE-IP to CBCT

In keeping with our departmental practice for online matching between CBCT and AVE-IP scans, the following steps were applied offline using Varian Offline Review. Firstly, an anatomical auto-match was applied in the region-of-interest using grayscale recognition. This was visually reviewed by a radiation therapist and radiation oncologist and corrected as required to ensure that vertebral bodies were aligned. Secondly, an auto-match to the liver edge was performed. Finally, a manual match was performed based on the RO beads contoured on the AVE-IP plan using grayscale blending, split windows, and contour overlay matching (Supplementary Appendix A2). Following matching based on RO beads, the centre of mass (CoM) for each RO bead area was compared between CBCT and AVE-IP in the mediolateral (ML), anteroposterior (AP) and cranio-caudal (CC) directions, and difference recorded. An absolute mean difference for each patient was calculated from all contoured RO bead areas. Finally, to compare RO bead position over time, bead position on 4D-CT data images (AVE-IP and CT-50 phase) was compared to bead position 6–20 days later on a diagnostic CT scan using the globally-optimal iterative closest point algorithm (Go-ICP).^23^ This process is outlined in Supplementary Appendix A3.

### RO beads in a phantom model

As patients did not undergo treatment with SBRT, in order to obtain further IGRT images of the RO beads, an in-house phantom was created. This process is outlined in detail in Supplementary Appendix A4. Static images of the RO bead phantom were first acquired to represent the RO beads in an end-exhalation phase, which included helical CT, kV, MV and CBCT scans. To replicate breathing motion, the phantom was placed on to the QUASAR^™^ Respiratory Motion Phantom (ModusQA, London, Ontario). This is a moving platform that can be programmed to simulate internal respiratory motion in the CC direction (detected on the radiotherapy images). There is a second smaller chest wall platform that can stimulate the external motion of the chest in the AP direction. The Varian RPM marker block was placed on the chest wall phantom to generate a surrogate signal for internal respiration during the non-static helical scans, 4D-CT and treatment simulations to allow phase sorting of the 4D CT images (Supplementary Appendix A5). The QUASAR^™^ phantom was set to a breathing cycle of 5 s, with 10 mm CC amplitude and the following obtained: helical CT scan, kV, MV and CBCT. RO bead contouring was performed using the same method as for the clinical scans.

## Results

### Patients

Between August 2017 and February 2019, eight patients were enrolled on to the VEROnA study: two patients with HCC and six with mCRC. Two patients had multiple tumours, and tumour diameter ranged from 8 to 82 mm. All patients underwent successful treatment with VERB. Six patients (75%) received the full volume (1 ml), whereas one patient received 0.4 ml and one patient 0.9 ml due to early stasis during the treatment procedure. 4D-CT and CBCT images were successfully acquired for all eight patients. For one patient, the primary radiotherapy data set was selected as the MIP as opposed to the AVE-IP, which subsequently excluded this patient from matching analysis (patient 4). Patient, tumour and treatment details are shown in Table 1.

**Table 1.** Baseline tumour, treatment and liver motion details. Liver motion during imaging was measured in the cranio-caudal direction based on the mid-dome of the liver on coronal slices of the 4D-CT images

Patient	Diagnosis	Number of lesions treated	Size of treated lesion (s) (mm)	Liver segment	Volume of VERB delivered (ml)	Liver motion (mm)
1	HCC	1	33	VIII	1	11
2	HCC	1	82	VII	1	12
3	mCRC	1	21	VII	1	14
4	mCRC	1	8	II/IVa	1	9
5	mCRC	1	12	VII	1	22
6	mCRC	1	40	V	0.4	14
7	mCRC	1	24	V	0.9	15
8	mCRC	3	42 + 29+16	IV	1	12

### Creation of fiducial markers from RO beads

RO beads were visible for all patients on the radiotherapy planning scans (AVE-IP image from 4D dataset) and CBCT imaging. The mean HU of the RO beads across all patients (for the three areas initially manually contoured) was 150 HU. Areas of RO beads were then successfully auto-contoured for all patients at 150 and 200 HU on the AVE-IP and CBCT scans; below 150 HU, areas of artefact were contoured by the automated thresholding and at 250 HU and above fewer than three discrete areas were contoured. For two patients (patients 5 and 7), areas of RO beads on the CBCT were unable to be separated into discrete areas due to the distribution of the beads along the vasculature. In these cases, the whole area was contoured for matching purposes. The median number of contoured RO bead areas on the CBCT at 150 HU was 3 (range 1–4), and 1.5 (range 0–4) at 200 HU. For the AVE-IP images, median number of contoured RO bead areas at 150 HU was 3 (range 0–7) and at 200 HU 1.5 (range 0–4) (Supplementary Appendix B1).

### Matching of RO beads

**Figure 3.**
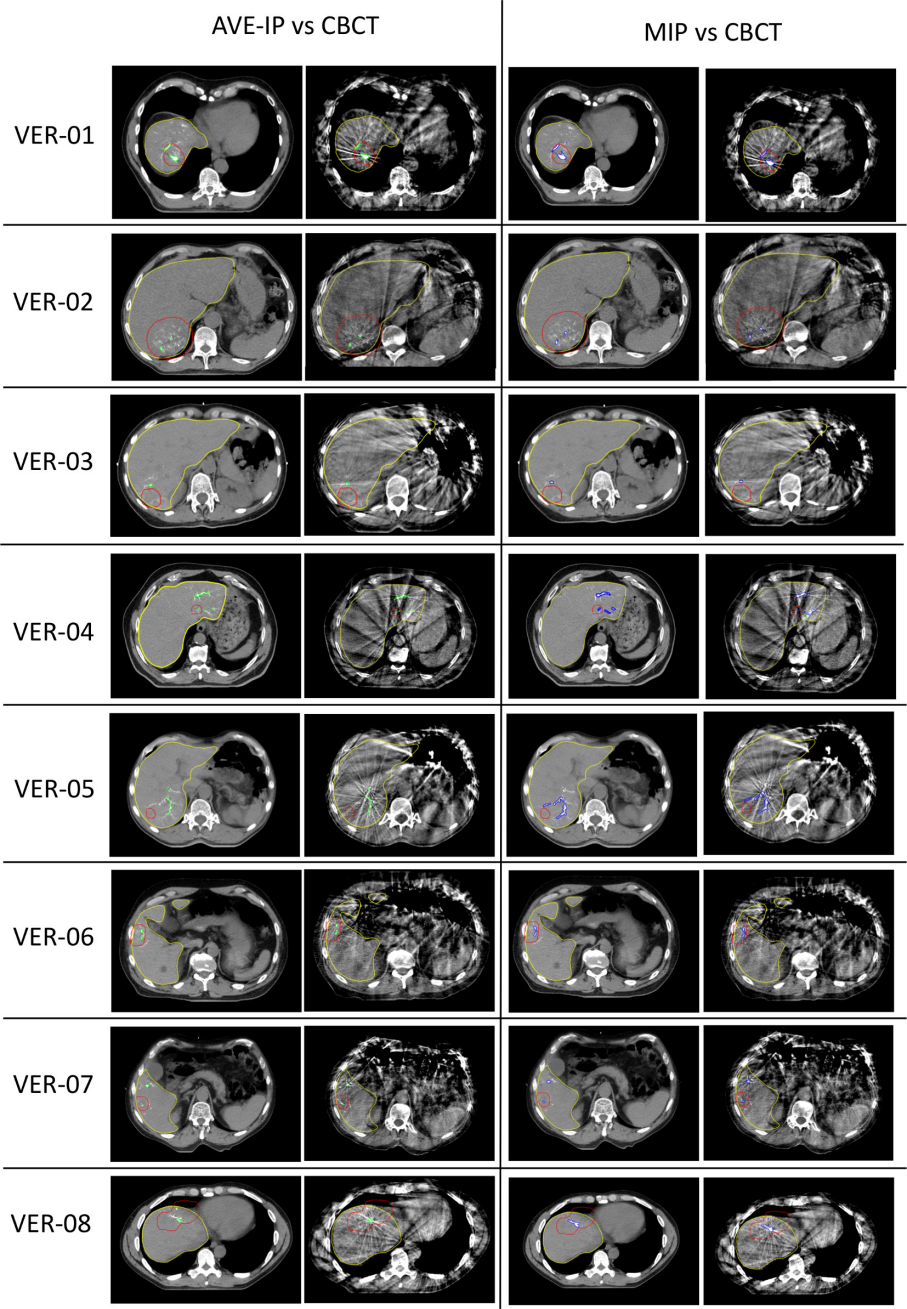
Matching of RO beads on AVE-IP, MIP and CBCT. Code: Red, planning target volume; Yellow, liver contour; Green, RO beads contoured on AVE-IP at 150 HU and matched to CBCT; Blue, RO beads contoured on MIP at 250 HU and matched to CBCT

Matching of CBCT to the radiotherapy planning scan (AVE-IP) images using areas of contoured RO beads was successful in all patients (Figure 3). Due to the optimal number of RO bead areas contoured for each patient using a threshold of 150 HU on CBCT and 4D-CT, these contours were used for final matching analysis. Matching was performed using six degrees of freedom. The difference in couch shifts between RO beads and bony anatomy (vertebral bodies) and RO beads and liver edge ranged from 1.9 to 4.0 mm (Supplementary Appendix B2).

After matching on RO beads, the absolute mean shift in CoM from CBCT to AVE-IP was 2.0 mm (SD 1.1) ML, 1.7 mm (SD 1.3) AP, and 3.5 mm (SD 2.4) CC (Supplementary Appendix B3). Figure 3 shows the final matching of AVE-IP to CBCT, and MIP to CBCT for all patients.

### Stability of RO bead position

**Figure 4.**
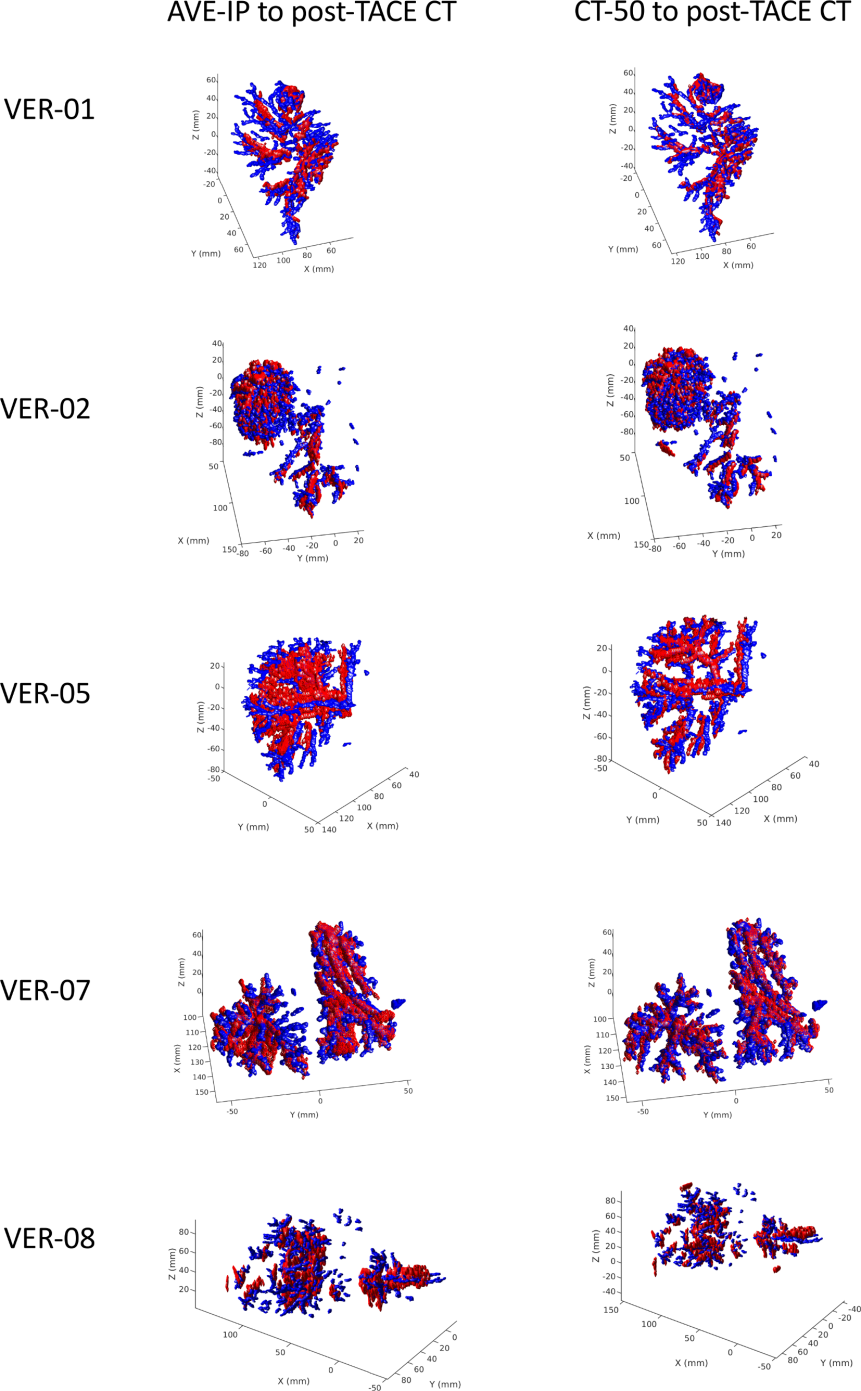
Comparison of bead position between 4D-CT images taken 1 day after treatment and CT scans taken post-TACE. AVE-IP and CT-50 scans are registered to the post-TACE CT scan using a Go-ICP algorithm.^23^ Red areas are the beads contoured on the 4D-CT scans (AVE-IP and CT50) and blue areas are the beads contoured on the post-TACE CT scans. Data for patient 3 are not shown as the field of view on the post-TACE CT did not encompass the entire liver. Patient 4 was excluded from this section of the analysis as the MIP was selected as the primary data set and not the AVE-IP. Patient 6 was excluded due to trapped contrast from the TACE procedure being present on the AVE-IP scan which impacted analysis

Comparison of bead position on 4D-CT scans (AVE-IP and CT-50) with post-TACE CT scans showed good alignment of beads along tumour vasculature up to 29 days later (Figure 4).

### RO bead phantom results

**Figure 5.**
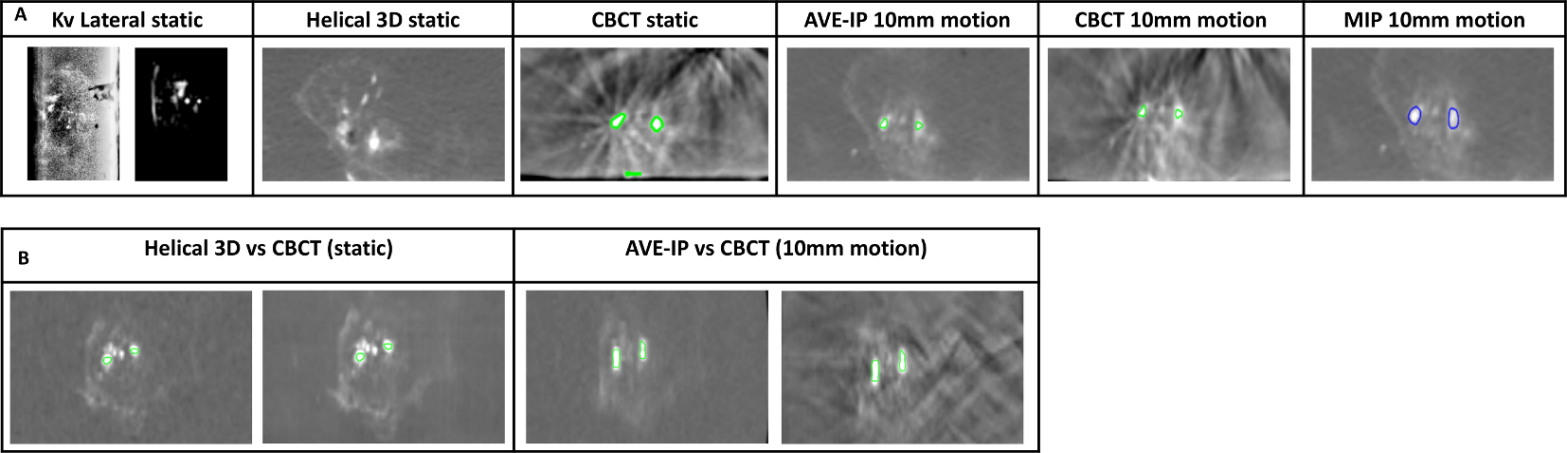
Radiopaque beads in a phantom model. A: Comparison of RO beads visibility with different imaging modalities. B: Change in shape of RO beads between static and motion images

RO beads were visible on all imaging modalities in both the static and moving phantom (Figure 5). Using automated threshold contouring at 150 HU, five discrete areas of RO beads were contoured on the CBCT images and three on the AVE-IP images. Absolute mean difference in CoM between areas of RO beads on static CBCT and AVE-IP images were 0.5 mm (SD 0.2) ML, 0.4 mm (SD 0.2) AP and 0.6 mm (SD 0.6) in the CC direction. When 10 mm of cranio-caudal motion was applied, translational errors were 7.4 mm (SD 1.7), 5.6 mm (SD 0.8) and 5.1 mm (SD 3.3) in the ML, AP and CC directions.

## Discussion

Drug-eluting beads are an effectively combined anti-cancer drug and embolisation treatment delivered directly to liver tumours during TACE treatment. The development of a novel RO drug-eluting bead, with durable radiopacity on CT scans, optimises the delivery technique by providing confirmation of bead location during and after the embolisation procedure.^20,21^ Based on the findings of this study, we propose that a TACE-SBRT combination is likely to become a therapeutic option, using RO drug-eluting beads that can function as potential surrogates of liver tumour targeting (fiducials) for IGRT.

In this study, we have demonstrated that RO beads are visible on the imaging modalities required for IGRT, including CBCT, 4D-CT, kV and MV. Furthermore, we have shown that on-treatment matching can be performed using RO beads using CBCT scans. Due to size of the RO beads, and their varied clumping and distribution along tumour vasculature, the shape and size of high-density regions in and around a tumour are not uniform. It is also apparent from the radiotherapy images that there is a difference between the distribution of beads between HCC and mCRC tumours, with the beads being localised in HCC tumours. However, the sample size for this study was small, so definite conclusions about localisation of beads cannot be drawn comparing HCC to liver metastases from CRC. As predicted, highly selective TACE delivery is possible with hypervascular HCC tumours, whereas mCRC tumours tend to be less vascular in nature, leading to a more lobar distribution of beads during TACE delivery. Despite this, using an automated contouring method based on HU thresholding, distinct areas of RO beads can be contoured on radiotherapy planning CT scans and accurately matched to areas of RO beads on CBCT imaging for both types of liver tumours. Without any formal motion mitigation, matching on CoM of contoured areas was possible within 3.5 mm. It is likely that with the application of motion mitigation methods, as used in standard clinical practice, this translational shift will be reduced further. As shown in the phantom study using static images, matching between CBCT and AVE-IP scans is possible within 0.6 mm. However, the impact of motion on the shape, and therefore CoM, of an irregular-shaped object is evident (Figure 5B). This is also demonstrated in patient 5, where liver motion was 22 mm and the difference in CoM of the RO bead area was 7.5 mm in the CC direction (Supplementary Table B3). When motion was applied to the phantom however, the error was greatest along the ML axis, which was not expected. When utilising 4D-CT for RT planning, patient-specific internal target volumes (ITV) can be created to minimise intrafraction errors. As with tumour contouring, in which the ITV is created by combining tumour volumes from all respiratory phases, this approach can also be applied to the RO beads, and a ‘fiducial ITV’ created that can be utilised for matching. The ability to match on CBCT, whether this is with an end-expiration phase, or AVE-IP, shows that RO beads can function as a surrogate for liver position in these approaches.

Although artefact streaking was present on CBCT, this did not impact RO bead visibility or the ability to match. Artefact streaking is a particular problem with commercial fiducials that can distort tumour contouring.^24^ Furthermore, artefact did not distort the ability to visualise or contour the tumour and, for HCC patients in whom RO beads were located within the tumour vasculature, tumour contouring was enhanced.

When RO bead matching was compared to matching based on liver edge or bony structures, difference in three-dimensional distances of up to 4.0 mm were seen. These findings suggest that bony anatomy and liver edge may be sub-optimal surrogates for tumour positioning in image- guided RT. This is in keeping with the findings by Yue et al, who compared matching on Lipiodol and bony anatomy; differences in three-dimensional distances were 0.9–2.6 mm (maximum 3.8 mm) in the ML direction, 1.1–2.9 mm (maximum 4.3 mm) in the AP direction, and 1.2–3.9 mm (maximum 5.5 mm) in the in the CC direction.^17^

There are limitations to our study and in the potential use of RO beads as fiducial markers. Firstly, this was a first-in-human clinical trial and, as such, our patient numbers are small and include just two patients with HCC. Furthermore, as patients did not undergo SBRT in this proof-of-principle study, radiotherapy imaging was limited to 4D-CT scans and CBCT images, and patients did not have any motion mitigation. As shown with the phantom model, translational errors and matching accuracy is improved with reduced motion. We also used an automated contouring method to contour RO beads. Although this approach was taken to reduce variation in contouring, this may not be available on all radiotherapy planning systems. However, areas of beads can be contoured manually without this approach. The use of RO beads as fiducials may also increase the complexity of RT planning and treatment delivery. Experience in contouring and matching on small areas of high intensity may take additional time. A final limitation of this study was that it did not specifically look at the impact of RO beads on dosimetry. Given the small volume of the high-density regions, it is anticipated that this will be negligible, but further evaluation would be required in a larger clinical study.

In conclusion, this study has shown that RO drug-eluting beads are visible on IGRT imaging modalities required for liver SBRT, show minimal artefact, can be reliably contoured, can be used for on-set matching with CBCT, and remain stable positionally within the liver vasculature. As such, their role as potential fiducial markers is feasible and warrants further exploration in combination studies of TACE followed by SBRT for liver tumours.
