# Supplementary material for: Radiopaque drug-eluting embolisation beads as fiducial markers for stereotactic liver radiotherapy
Source: Br J Radiol. 2021 Nov 16;95(1130):20210594. doi: 10.1259/bjr.20210594 (PMC8822567; doi:10.1259/bjr.20210594)
Supplement: Supplementary Material. [file bjr.20210594.suppl-01.docx]

**APPENDIX A**

**Figure A1: Flow diagram of contouring methodology**

**
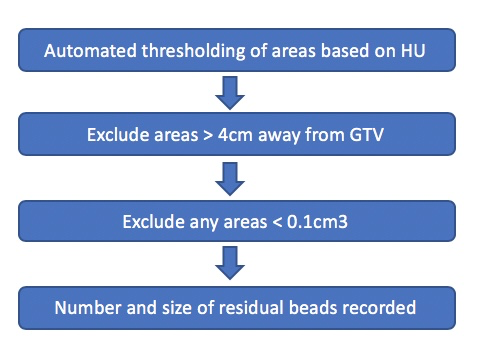
**

**Figure A2: a) Vertebral matching with gray scale, b) Liver contour matching, c) RO bead matching**


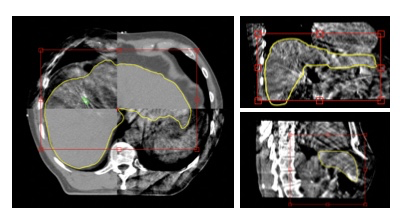

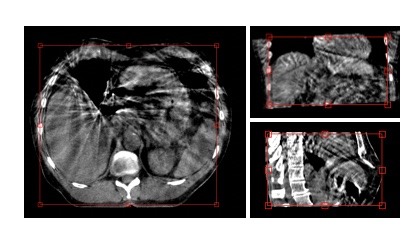

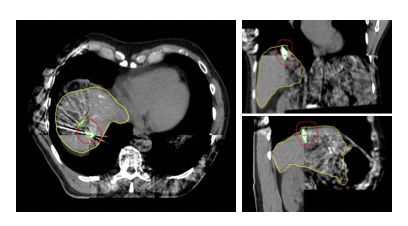


**A3: Position of RO beads after placement.**

In order to compare RO bead position over a period of time, the bead position on 4D-CT was compared to bead position on CT imaging 6-20 days later. The AVE-IP and CT 50 scans for each patient were matched to the diagnostic CT scan using an in-house rigid registration method. In brief, the RO beads were contoured on MATLAB® using automatic thresholding set to 2 SD above the mean liver region (HU). Segmentation was then refined with a local threshold (2 SD of background above the mean) limited to the 5 mm region around the vasculature and small connected components were filtered out to produce final segmentations of the embolised vasculature. Surface meshes were generated for each segmentation and nodes of these surfaces registered to each other using the globally-optimal iterative closest point algorithm (Go-ICP).

**A4: Creation of phantom for RO beads with a central tumour.**

In brief, a simple phantom was created by filling a 2-litre plastic box with 2.5% liquid agar. The 2.5% agar provided a background Hounsfield unit range closest to that of liver on CT imaging. In order to create a RO bead phantom, a 4 cm circular sponge was first filled with agar in a cylindrical shell (A). Once solidified this was placed into a box filled with liquid agar (B). When the liquid agar was in a semi-solid state (to prevent the development of air gaps) 1 mL of the RO beads in 9ml of saline was injected into the sponge in order to align the RO beads in a distribution that was comparable to tumour vasculature. The beads were mixed with 9 mL of saline to represent bead delivery in the clinical situation, in which the beads are mixed with 9mL of Omnipaque for TACE delivery. A 22 G catheter and three way-tap was utilised to insert the beads into the tumour to avoid bead clumping. The phantom was then left to cool and solidify prior to imaging.

**Phantom creation**

A: Agar filled sponge placed within a circular shell in order to create a 4 cm tumour

B: 4 cm tumour placed within a box filled with liquid agar.

**
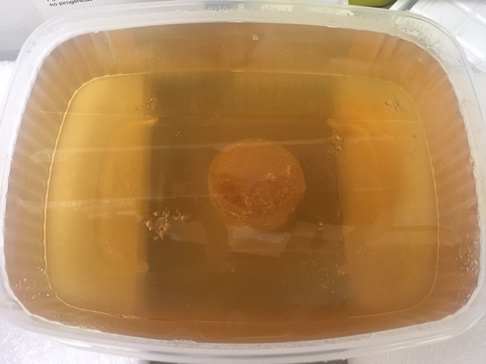

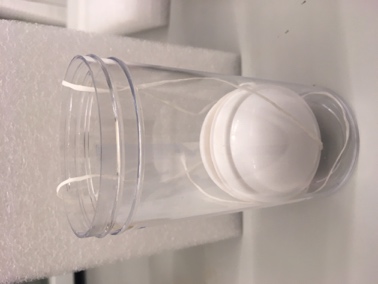
**

**A5: QUASAR phantom**

Image of the QUASAR™ Respiratory Motion Phantom (ModusQA, London, Ontario)

**
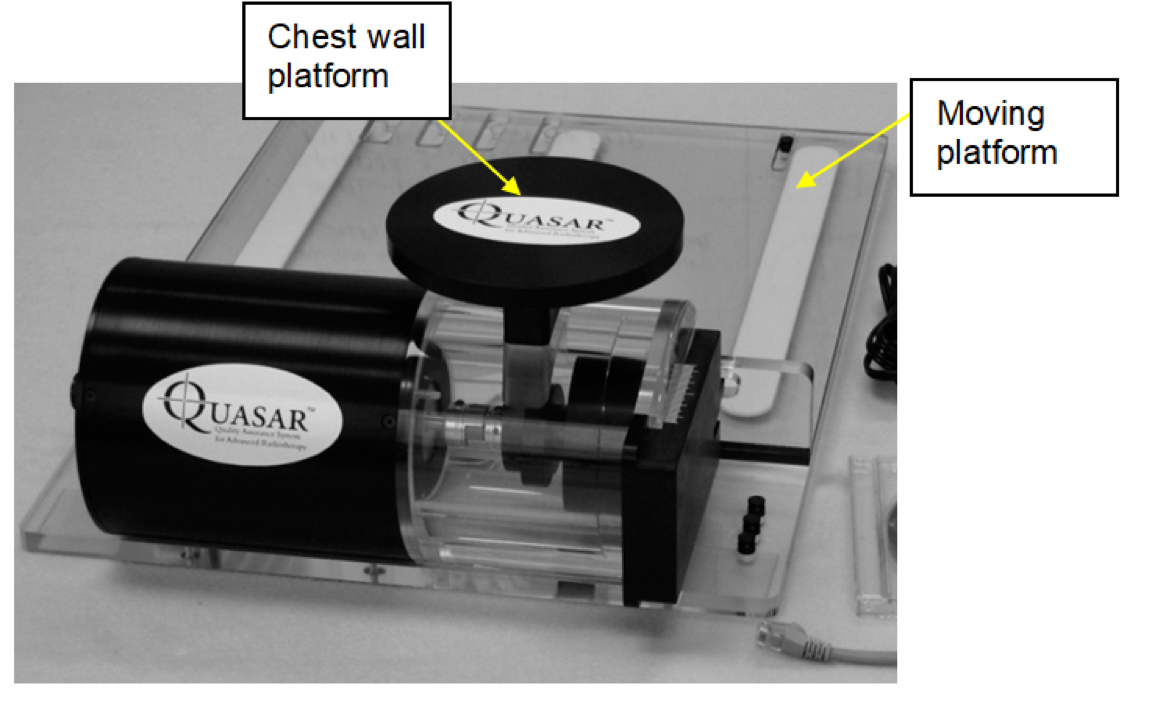
**

The QUASAR phantom used consists of two moving platforms: there is a moving platform that can be programmed to simulate internal respiratory motion in the CC direction (detected on the radiotherapy images); and a smaller chest wall platform which can simulate the external motion of the chest in the AP direction.

**APPENDIX B**

**Table B1. Contoured RO bead areas on CBCT and AVE-IP images**

*For patients 5 and 7 only one area of RO bead is contoured due to an inability to separate the RO beads into discrete areas due to the distribution of the beads. Zero (0) represents the situation where no areas of RO beads > 1cm^3^ were contoured at that Hounsfield unit (HU) threshold.

|  | 1 | 2 | 3 | 4 | 5 | 6 | 7 | 8 |
| --- | --- | --- | --- | --- | --- | --- | --- | --- |
| **CBCT 150 HU** |  |  |  |  |  |  |  |  |
| Number of areas | 3 | 3 | 1 | 3 | 1* | 3 | 1* | 4 |
| Total volume (cm^3^) | 1.9 | 0.3 | 0.2 | 1.6 | 2.2 | 0.8 | 1.7 | 4.8 |
| Volume of each area (cm^3^) | 1.7  0.1  0.1 | 0.1  0.1  0.1 | 0.2 | 0.8  0.1  0.7 | 2.2 | 0.1  0.2  0.5 | 1.7 | 1.1  3.1  0.5  0.1 |
| **CBCT 200 HU** |  |  |  |  |  |  |  |  |
| Number of areas | 1 | 0 | 0 | 2 | 1 | 2 | 4 | 3 |
| Total volume (cm^3^) | 1.45 |  |  | 0.5 | 0.5 | 0.2 | 0.7 | 2.0 |
| Volume of each area (cm^3^) | 1.45 |  |  | 0.3  0.2 | 0.5 | 0.1  0.1 | 0.1  0.1  0.4  0.1 | 0.4  1.4  0.2 |
| **AVE-IP 150** |  |  |  |  |  |  |  |  |
| Number of areas | 5 | 7 | 3 | 3 | 1* | 3 | 5 | 3 |
| Total volume (cm^3^) | 2.4 | 0.9 | 0.5 | 2.3 | 2.2 | 2.7 | 1.5 | 1.0 |
| Volume of each area (cm^3^) | 2.0  0.13  0.1  0.13  0.1 | 0.1  0.1  0.1  0.3  0.1  0.1  0.1 | 0.2  0.1  0.2 | 1.2  0.2  0.9 | 2.2 | 0.2  1.4  1.1 | 0.1  0.7  0.1  0.5  0.1 | 0.1  0.8  0.1 |
| **AVE-IP 200** |  |  |  |  |  |  |  |  |
| Number of areas | 1 | 1 | 0 | 3 | 4 | 2 | 2 | 1 |
| Total volume (cm^3^) | 1.6 | 0.1 |  | 0.8 | 0.8 | 0.4 | 0.5 | 0.8 |
| Volume of each area (cm^3^) | 1.6 | 0.1 |  | 0.5  0.1  0.2 | 0.1  0.3  0.1  0.3 | 0.3  0.1 | 0.4  0.1 | 0.8 |

**Table B2. Couch shifts from fiducials for bony and liver edge matching.**

No results available for patient 4 as the maximum intensity projection (MIP) series was selected as the primary series as opposed to the average intensity projection (AVE-IP) series. This patient was excluded from the matching analysis.

|  | **Bony match** | | **Liver Edge** | |
| --- | --- | --- | --- | --- |
|  | **Absolute**  **Mean shift** | **SD** | **Absolute**  **Mean shift** | **SD** |
| Mediolateral (mm) | 1.9 | 1.2 | 2.0 | 0.8 |
| Anteroposterior (mm) | 3.8 | 3.3 | 3.3 | 2.0 |
| Craniocaudal (mm) | 3.2 | 2.7 | 4.0 | 3.3 |
| Pitch^0^ | 1.24 | 1.55 | 1.67 | 1.65 |
| Roll^0^ | 2.63 | 2.92 | 2.61 | 2.76 |
| Rotation^0^ | 2.59 | 1.60 | 2.36 | 1.21 |

**Table B3: Absolute mean difference in centre of mass of fiducials from CBCT to AVE-IP post matching.**

No results available for patient 4 as the maximum intensity projection (MIP) series was selected as the primary series as opposed to the average intensity projection (AVE-IP) series. This patient was excluded from the matching analysis

|  | **1** | **2** | **3** | **5** | **6** | **7** | **8** | **Absolute Mean (SD)** |
| --- | --- | --- | --- | --- | --- | --- | --- | --- |
| Change in volume (cm^3^)  (% change) | 0.10  (5) | 0.10  (33) | 0.00  (0) | 0.3  (14) | 0.67  (84) | 0.13  (2) | 1.20  (25) | 0. 34 (0.41)  23 (25) |
| Mediolateral (mm) | 2.7 | 0.3 | 1.1 | 2.8 | 2.8 | 0.9 | 3.4 | 2.0 (1.1) |
| Anteroposterior, (mm) | 1.2 | 0.4 | 0.8 | 2.6 | 0.8 | 2.0 | 4.3 | 1.7 (1.3) |
| Craniocaudal, (mm) | 1.5 | 2.5 | 0.00 | 7.3 | 5.5 | 5.3 | 2.5 | 3.5 (2.4) |
